# Temperature-induced variation in gene expression burst size in metazoan cells

**DOI:** 10.1186/s12867-015-0048-2

**Published:** 2015-11-25

**Authors:** Ophélie Arnaud, Sam Meyer, Elodie Vallin, Guillaume Beslon, Olivier Gandrillon

**Affiliations:** Centre de Génétique et de Physiologie Moléculaire et Cellulaire (CGPhiMC), CNRS UMR5534, Université de Lyon, Université Lyon 1, 69622 Lyon, France; Laboratoire d’InfoRmatique en Image et Systèmes d’information (LIRIS), CNRS UMR5205, INSA-Lyon, INRIA, Université de Lyon, 69621 Lyon, France; Inria Team Dracula, Inria Center Grenoble Rhône-Alpes, Montbonnot-Saint-Martin, France; Inria Team Beagle, Inria Center Grenoble Rhône-Alpes, Montbonnot-Saint-Martin, France; Division of Genomic Technologies, RIKEN Center for Life Science Technologies, Yokohama, Japan; INSA-Lyon, CNRS UMR5240 Microbiologie, Adaptation et Pathogénie, Université de Lyon, 69622 Lyon, France; Laboratoire de Biologie Moléculaire de la Cellule, Ecole Normale Supérieure de Lyon, CNRS, Université de Lyon, 46 Allée d’Italie, 69007 Lyon, France

**Keywords:** Expression noise, Stochastic model, Temperature

## Abstract

**Background:**

Gene expression is an inherently stochastic process, owing to its dynamic molecular nature. Protein amount distributions, which can be acquired by cytometry using a reporter gene, can inform about the mechanisms of the underlying microscopic molecular system.

**Results:**

By using different clones of chicken erythroid progenitor cells harboring different integration sites of a CMV-driven mCherry protein, we investigated the dynamical behavior of such distributions. We show that, on short term, clone distributions can be quickly regenerated from small population samples with a high accuracy. On longer term, on the contrary, we show variations manifested by correlated fluctuation in the Mean Fluorescence Intensity. In search for a possible cause of this correlation, we demonstrate that in response to small temperature variations cells are able to adjust their gene expression rate: a modest (2 °C) increase in external temperature induces a significant down regulation of mean expression values, with a reverse effect observed when the temperature is decreased. Using a two-state model of gene expression we further demonstrate that temperature acts by modifying the size of transcription bursts, while the burst frequency of the investigated promoter is less systematically affected.

**Conclusions:**

For the first time, we report that transcription burst size is a key parameter for gene expression that metazoan cells from homeotherm animals can modify in response to an external thermal stimulus.

**Electronic supplementary material:**

The online version of this article (doi:10.1186/s12867-015-0048-2) contains supplementary material, which is available to authorized users.

## Background

Gene expression is an inherently stochastic process, owing to its molecular nature [1]. During the last 15 years, stochasticity in gene expression has been extensively studied and it has become clear that it plays a crucial role in numerous physiological processes (see [2] for a recent review). The first studies providing evidence of stochasticity in gene expression have been conducted on prokaryotic organisms [3, 4]. Then, experiments conducted on eukaryotic organisms indicated that the causes of stochasticity could differ between prokaryotes and eukaryotes [5–8]. A number of mechanisms which influence the amount of stochasticity affecting a given gene have been identified (see [2]), ranging from chromatin dynamics [9, 10] to network dynamical architecture [11, 12].

In order to analyze gene expression experimental data, it is useful to introduce mathematical models of the expression process. In the classical “two-state model” [13, 14], a gene switches from a closed to an open state with constant rates. Although simplified, this description is relevant enough to allow reproducing many features of stochastic expression data, and to infer the underlying chromatin dynamics. In particular, it is able to describe the eukaryotic bursty transcription regime, where the gene is mostly closed and opens only for brief periods of times. Moreover, this model is simple enough to be fitted on high-throughput data such as fluorescence distributions measured by cytometry. Using this approach, we recently showed that the genomic position strongly affects the frequency of bursts, rather than burst size. We demonstrated that differences in chromatin dynamics at the insertion site could explain at least part of the observed clone-to-clone variations [9].

It has been shown in numerous systems that stochasticity in gene expression was a driving force that allowed a given clone to reacquire its entire distribution [15–18], starting from as little as a single cell in a matter of days [19]. One open question then concerns the very long time stability (weeks to month) of the original distribution. In the present work, we used clones with different insertion sites of a fluorescent reporter under the control of the CMV promoter. The choice of such a system presents several advantages in our study. First, the CMV promoter being a viral promoter, the gene regulation network does not affect its transcription process. The same is true with the fluorescent reporter that has no biological effect and so will not be targeted from gene regulation networks. Moreover, we have previously characterized the CMV promoter behavior during chromatin remodeling [9] and we can thus use our validated model to study the dynamics of gene expression. Importantly, CMV is an exogenous promoter: thus, even though the results presented might be limited to this specific promoter, the mechanisms involved in its regulation are likely not gene-specific, and may thus have a widespread relevance in genomic regulation.

In our study, the fluorescence of these clones has been recorded over more than 8 months. At such a long time scale, we surprisingly observed the existence of a correlated variation simultaneously affecting the mean expression of our different clones. We tested possible causes for such a clone-to-clone correlation and demonstrated that small temperature variations strongly affect the mean expression value while the normalized variance remains mostly unchanged. In particular, a 2 °C increase of temperature resulted in a 40 % reduction of fluorescence activity while a 2 °C decrease resulted in ~65 % augmentation of the same. By fitting a two-state model on the fluorescence distributions measured under different temperature conditions, we demonstrated that this effect was related to a modification of the burst size (i.e. the number of proteins produced during a transcription burst), while their frequency was less systematically altered. Altogether our data point toward external temperature influence as a possible cause in the generation of gene expression variations in metazoan cells from homeotherm animals.

## Methods

### Cell culture

All experiments were performed on stably transfected 6C2 cell clones that have been characterized previously [2, 9] and display a single integration site in their genome of a mCherry reporter gene under the control of a CMV promoter. Briefly, 6C2 cells are a chicken erythroblast cell line transformed by the avian erythroblastosis virus (AEV; [20]). They were generated 30 years ago in the lab headed by Dr. Hartmut Beug. At that time there was no animal ethics committee. We did not do any animal work by ourselfes, and obtained the cell line from our colleague. 6C2 are cultured at 37 °C under 5 % CO_2_ in α-minimal essential medium (Gibco) supplemented with 10 % (v/v) fetal bovine serum, 1 % (v/v) normal chicken serum, 100 µM β-mercaptoethanol (Sigma-Aldrich), 100 units/ml penicillin and 100 μg/ml streptomycin (Gibco).

### Flow cytometry

The mCherry expression level and the cell size were measured by flow cytometry with a FACS canto II (Beckson Dickinson).

Regarding the mCherry fluorescence, the cells were seeded at a fixed concentration 24 h before their analysis. Regarding the cell size, the cells were seeded at fixed concentration 5 days before their analysis and incubated at different temperature. The measurement of the Side Scatter Area (SSC-A), considered as a proxy for the cell volume [21], has been used to estimate the cell size. The day of the analysis, the cells were pelleted by centrifugation (200*g*, 5 min), suspended in Phosphate Buffer Saline 1× (PBS, Gibco) and kept in dark on ice until their analysis.

The mCherry expression and the cell size were determined on 50,000 living cells, gated with the Flowjo software. The stability of the cytometer was checked and taken into account by analyzing, in each experiment, flow calibration particles (SPHEROTM Rainbow; Spherotech Inc. Lake Forest, IL, USA) as a calibration reference.

### Recovery experiments

The following amount of cells was sorted by FACS among the 10 % of cells with the highest mCherry expression rate: 10,000, 1000, 100 and 10 cells. Those cells were seeded in the following volumes: 10, 1, 0.1 and 0.01 ml so as to keep the seeding concentration constant. Cells were grown and analyzed as soon as we obtained at least 50,000 living cells.

### Statistical analysis

All statistical analyses were performed with the R software [22]. Correlation analyses were conducted with the Spearman correlation coefficient, and a limiting *p* value of 0.05. Fluorescence distributions were characterized by their mean fluorescence intensity (MFI) and normalized variance (NV). Regarding the effect of the temperature or CO_2_ on the fluorescence, cells size and cell division, all statistical analyses were performed on relative values, where the data was first normalized by the values obtained in standard cell culture condition on the same clone. This representation allows a better comparison of the results as the MFI and the NV could be very different depending on the transgene insertion site. To compare the samples in different conditions, we employed paired Wilcoxon tests, with a threshold of 0.05 on the p value.

### Fluorescence measurements on fixed cells

In order to assess whether or not the observed variations in cell fluorescence could be due to variation in intrinsic protein fluorescence intensity, cells were grown at 37 °C, fixed in 4 % paraformaldehyde in PBS, rinsed twice in PBS, and incubated for 24 h at 35, 37 or 39 °C before flow cytometry.

### Protein and mRNAs half-life measurements

Proteins and mRNAs half-life was measured as previously described [9]. Briefly, to determine the mRNA half-life, the cells were first treated with actinomycin D and the mRNA concentration was measured with a quantitative-reverse-transcription-Polymerase Chain Reaction (qRT-PCR) assay. Regarding the mCherry half-life, the cells were first treated with the cycloheximide and the mCherry concentration was determined by flow cytometry.

### Two-state model of gene expression

**Fig. 1:**
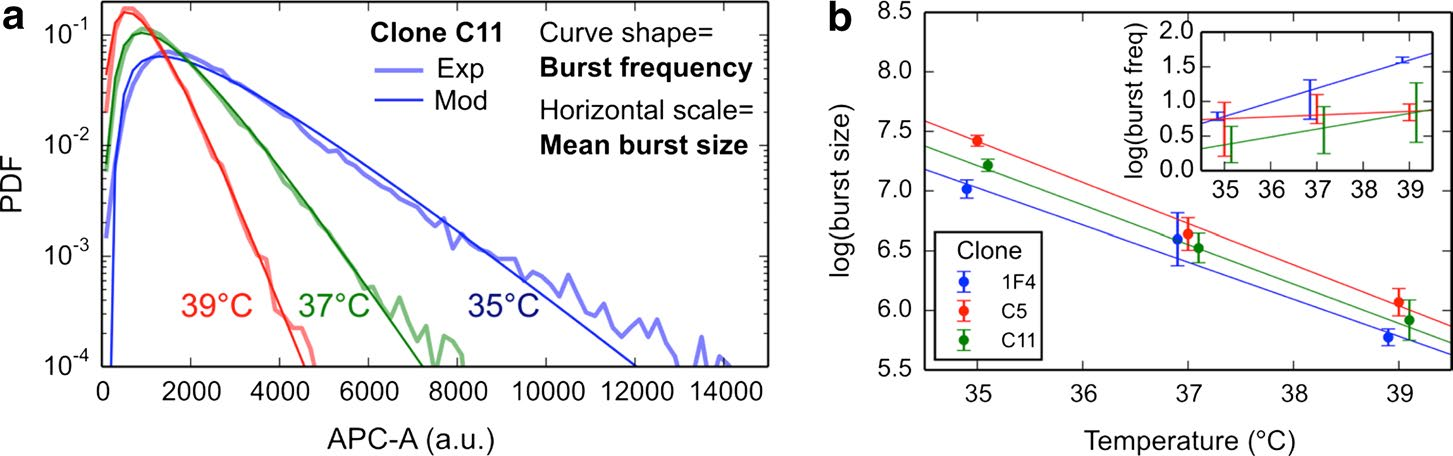
Inference of the bursting kinetic parameters from the experimental distributions after 120 h incubation. **a** The *curves* are fitted by negative binomial distributions, where the *curve shape* is related to the burst frequency and the *curve horizontal* scale to the mean number of proteins per burst (see “Methods”). The burst size is the parameter mostly affected by temperature. **b** The comparison of the parameters inferred on different clones suggests that the burst frequency and size depend on the insertion site, but with a common temperature-dependence of the burst size (slope of the lines), probably indicative of the same reaction network involved in gene expression, and characterized by an effective activation energy E_A_ = −60 ± 3 kcal/mol. The burst frequency exhibits a limited, clone-dependent increase. Values on *vertical axes* are expressed in natural logarithm

We previously showed [9] that in the considered system, protein distributions are well reproduced by a two-state model of gene expression, where mRNAs are produced during short transcription bursts (“on” state), separated by long inactive periods (“off”). Here, we took advantage of this behavior to simplify the fitting procedure by considering the limit of infinitely short bursts (i.e. by considering that for a given transcription burst, all mRNAs are produced simultaneously). The formalism is then mathematically equivalent to a “one-state model” (Eq. 8 in [23]), except that the burst duration is given by 1/k_off_ rather than the RNA lifetime. This approximation is relevant here since the mCherry reporter protein half-life is much longer than (1) the burst duration and (2) the RNA half-life, and it is further validated by the good agreement of the model with experimental curves (Fig. 1a). In this case the fluorescence distribution is a negative binomial [23] that be computed analytically. It only depends on two parameters: the burst size (*b*; number of proteins produced per burst) that gives the distribution shape and the burst frequency normalized by the protein lifetime (*f*) that gives the scale of the distribution:

$$b = \frac{\rho \gamma }{{ \tilde{\rho }k_{\text{off}} }}\quad f = \frac{{k_{\text{on}} }}{{\tilde{\gamma } }}$$

where $\rho$ and $\tilde{\rho }$ are the RNA production and degradation rates, $\gamma$ and $\tilde{\gamma }$ the protein production and degradation rates, and $k_{\text{on}}$ and $k_{\text{off}}$ the burst on/off switching rates, respectively (see [9] for details). These two parameters of the model curves were fitted on the logarithmic distributions with a least-squares method, with a small additional free translational parameter accounting for the background fluorescence. We determined the two degradation rates in independent experiments (see above and “Results”). Since in our procedure $\rho$, $\gamma$ and $k_{\text{off}}$ parameters cannot be separated, we aggregate them into a single parameter $\rho \gamma /k_{\text{off}}$.

### Temperature dependence of kinetic parameters

We describe the temperature dependence of the computed kinetic parameters in analogy to classical kinetic theories [24]. A reaction-limited elementary process is characterized by an activation energy $E_{A}$, which can be estimated from an Arrhenius plot ($log(k)$ vs. $1/T$). For small temperature variations around $T_{0}$, $\varDelta T \ll T_{0}$ this energy can be simply computed from the slope of the graph $log(k)$ vs. $T$ (Fig. 1b): $log(k(T_{0} + \varDelta T)) = log(k(T_{0} )) + (E_{A} /k_{B} T) \cdot (\varDelta T/T_{0} )$. For diffusion-limited reactions, the same graph would yield a slope corresponding to the case $E_{A} = k_{B} T$. Importantly, in both regimes, the kinetics increases with temperature. Here, the burst processes likely involve a complex combination of elementary processes, and the resulting temperature dependence could thus be increasing or decreasing. We compute “effective activation energies” for the inferred parameters, in analogy to the classical theories, and later discuss the signification of these quantities in terms of molecular events. Importantly, for combined kinetic processes [e.g. $k = \left( {k_{1} \cdot k_{2} /k_{3} } \right)$], the total effective activation energy is the sum of the single ones: $E_{A} = E_{A1} + E_{A2} - E_{A3}$. In contrast to the usual version, the effective activation energy can thus be negative if the reaction is inversely dependent on the limiting process.

## Results

### Characteristic relaxation time

Our first goal was to establish how fast an isolated fraction of cells could recapitulate their initial distribution. For this, we sorted cells from a fluorescent reporter-expressing clone in four sub-populations with different sizes, keeping the cell concentration constant (see “Methods”). The cells were then incubated and analyzed by flow cytometry as soon as the number of cells was sufficient: this took 3 days for the larger cell sub-populations and up to 9 days for the smallest cell sub-populations. All the sub-populations had returned to their original distribution by the time we could perform the first flow cytometry analysis. This indicated that the original distribution was a robust phenotype for a given clone, and that cells regained this stable regime very fast. We then wondered how stable this phenotype might be under much longer observation periods.

### Fluorescence variations over a long period

We decided to investigate the long-term behavior of two clones, C5 and C11. A third clone, 1F4, was added later in the experiment. At day 0, cells were randomly split in three sub-populations, a, b and c. The day before flow cytometry analysis, the cells of the sub-populations were plated at the same concentration, thereby avoiding possible variations induced by variations in cell concentration.

**Fig. 2:**
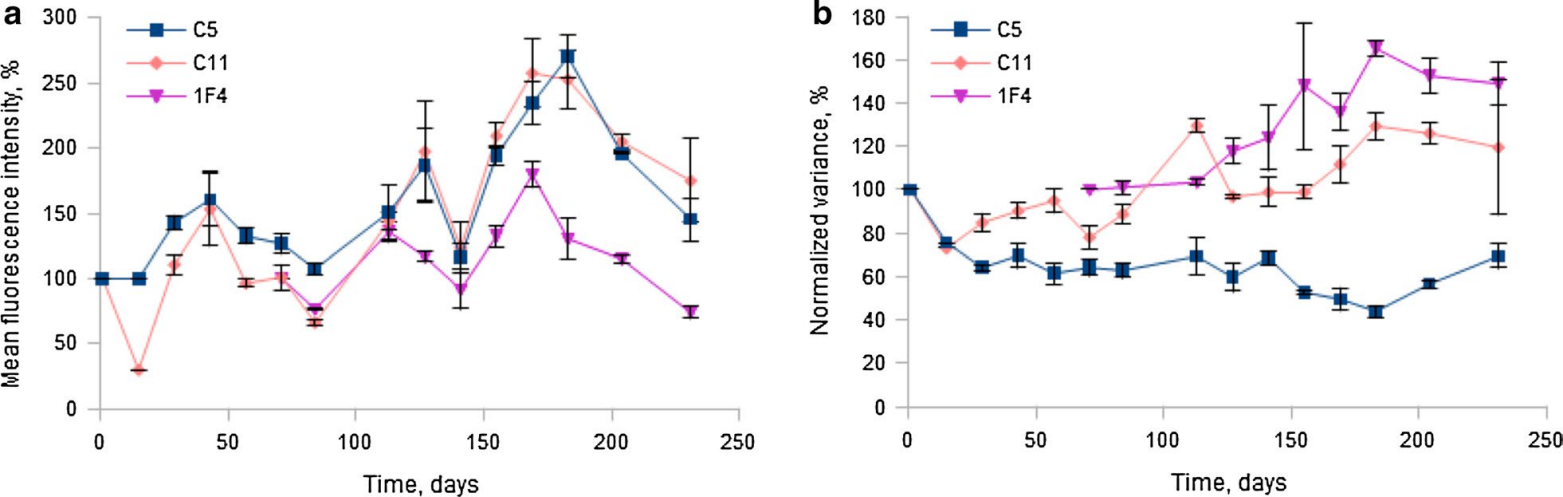
Variation of the mean fluorescence intensity (MFI) (**a**) and normalized variance (NV) (**b**) of mCherry during time. Three clones, randomly separated in three sub-populations at day 0, have been analyzed by flow cytometry. The MFI (**a**) and NV (**b**) were measured on 50,000 gated lived cells. *Each curve* represents the mean of the three sub-populations and the *errors bars* represent the standard deviation of the sub-populations

**Fig. 3:**
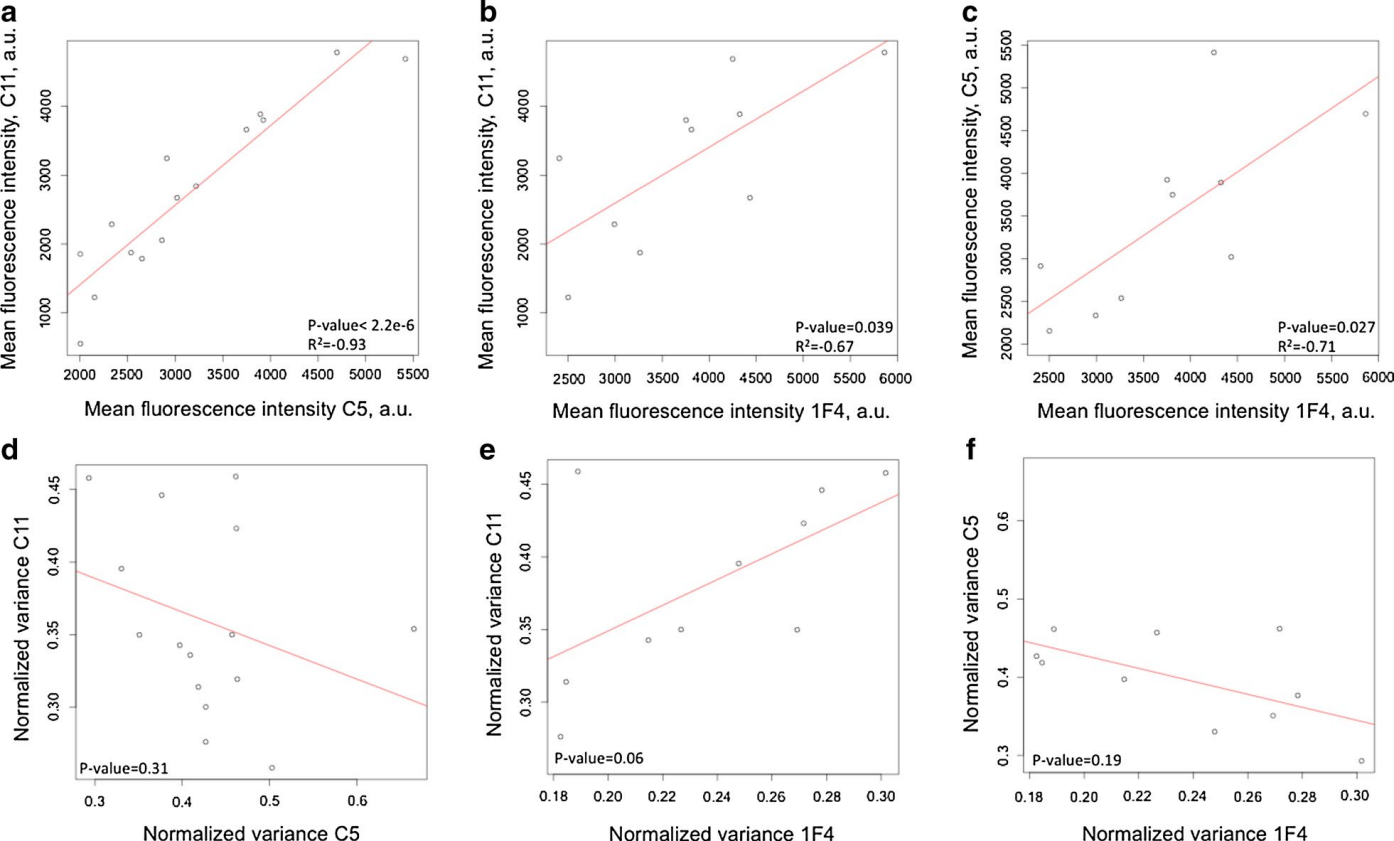
Correlation test of the mean fluorescence intensity (MFI) and the normalized variance (NV) of mCherry. Shown is the inter-clone correlation of the MFI (**a**–**c**) and NV (**d**–**f**). All clones are compared by pairs with a Spearman’s rank correlation test. When the p value was significant (p value <0.05), the correlation coefficient is indicated (R^2^)

We observed that the three sub-populations of each clone exhibited a remarkably similar mean fluorescence intensity (MFI) and normalized variance (NV) values at each given time point during the 230 days of the analysis (Additional file 1: Figures SD1 and SD2), confirming the robustness of the observed phenotypes. On the other hand, the MFI value of each clone exhibited strong time-dependent variations (Fig. 2a), while the normalized variance seemed to fluctuate on a less marked fluctuation range (Fig. 2b): the C11 MFI vary from 50 to 250 % whereas the C11 NV vary from 70 to 130 %, and the same holds true for the two others clones. Unexpectedly, the MFI variations of all clones appeared to be correlated (Fig. 2a), which was demonstrated by a Spearman correlation analysis (Fig. 3a–c). The normalized variances were not significantly correlated (Fig. 3d, e).

These results suggest that the MFI varies quite significantly during very long periods, whereas the NV value is more constant for a clone. Moreover, we clearly detected a correlation between MFIs, which points toward an external cause that would apply to all clones simultaneously. We therefore explored the influence of two possible environmental conditions, CO_2_ and temperature.

### External influences on gene expression

In order to test the impact of these two environmental parameters on mCherry expression, cells were incubated in several conditions and the MFI and NV of the reporter expression were determined by flow cytometry.

**Fig. 4:**
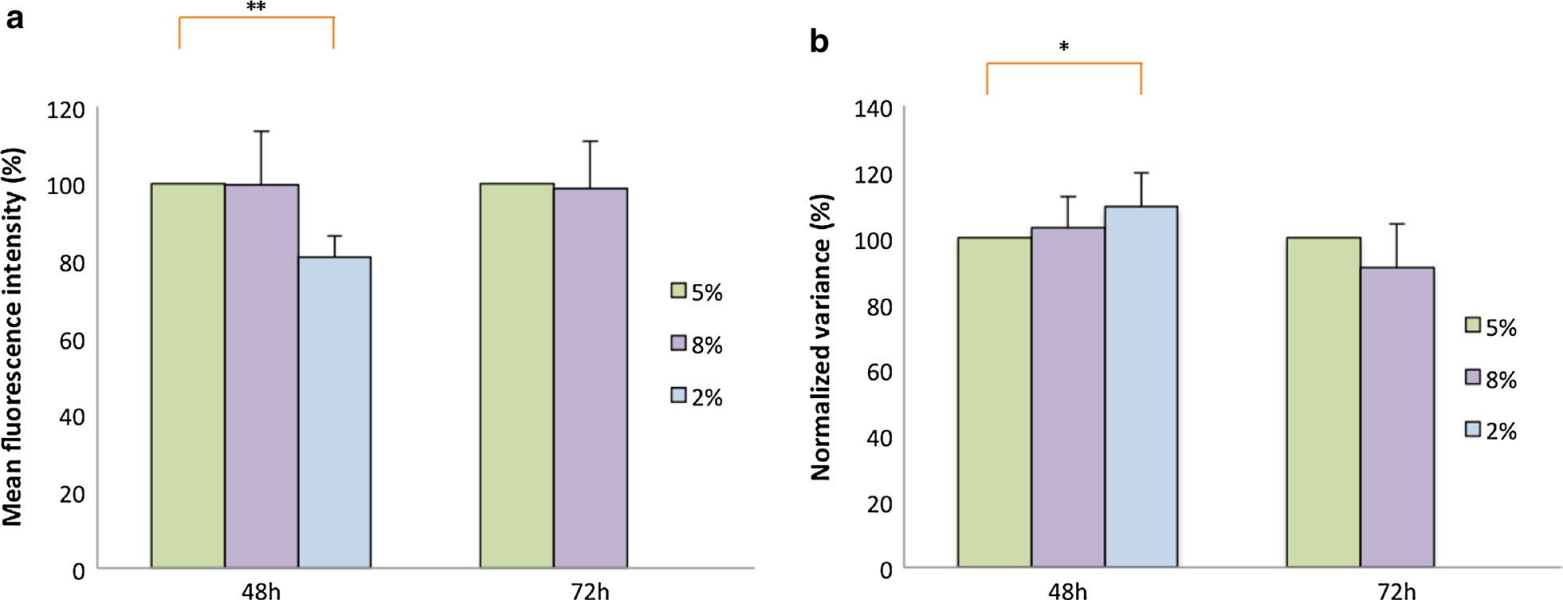
CO_2_ influence on the mean fluorescence intensity (MFI) and the normalized variance (NV). Cells were incubated at 5 % of CO_2_ as a standard concentration and at lower (2 %) or higher (8 %) concentration during 48 or 72 h. Then, the MFI (**a**) and the NV (**b**) were measured by flow cytometry on 50,000 gated lived cells. The raw data were transformed in percentage (see “Methods”). N = 9, *error bars* represent SD. *Brackets* indicate significantly different values (*p value <0.05; **p value <0.01 for a paired Wilcoxon test)

We tested the impact of a decrease (2 % of CO_2_ versus 5 % of CO_2_ in standard conditions) and an increase (8 % of CO_2_) of CO_2_ concentration. As shown in Fig. 4a, both types of changes in CO_2_ concentration induce a decrease of the MFI level, and no increase is observed. Conversely, for the NV level (Fig. 4b), no decrease was observed. Thus, these changes cannot account for the correlated MFI variations observed in Fig. 2a (MFI varied from 50 to 250 %, e.g. C11), nor for the NV variations observed in Fig. 2b.

**Fig. 5:**
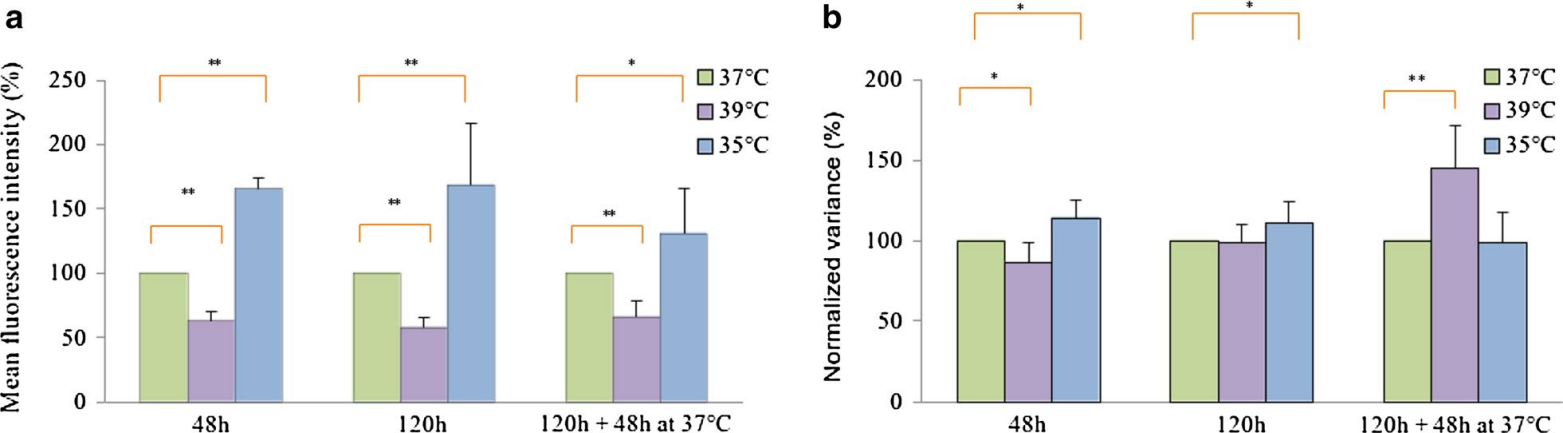
Temperature influence on the Mean fluorescence intensity (MFI) and the normalized variance (NV). Cells were incubated at indicated temperature during 48 or 120 h. Then, the cells were replaced at 37 °C during 48 h. The MFI (**a**) and the NV (**b**) were measured by flow cytometry on 50,000 gated lived cells. The raw data were transformed in percentage of the corresponding sample at 37 °C (see “Methods”). This representation permits a better comparison of the results as the MFI and the NV could be very different depending on the genome transgene insertion site. N = 9, *error bars* represent SD. *Brackets* indicate significantly different values (*p value <0.05; **p value <0.01 for a paired Wilcoxon test)

Regarding temperature, we also tested an increase (39 vs 37 °C in normal cell culture conditions) or a decrease (35 °C) of this parameter. It became immediately clear that temperature variation had a profound and significant influence on the mean value of the reporter distribution (Fig. 5a) whereas it had only a limited impact on its NV (Fig. 5b). A temperature increase from 37 to 39 °C induced a reduction of 37 % in 48 h and 43 % in 120 h of the mCherry MFI (Fig. 5a). When these cells were replaced at 37 °C, the MFI increased back to a level 33 % lower than initially, indicating a tendency to recover the initial distribution. The opposite was true when the culture temperature was decreased: after 48 h at 35 °C, the mCherry MFI value was higher by 64 and 68 % after 120 h; and the effect was back to 30 % after the cells had been replaced at 37 °C (Fig. 5a).

We noticed that temperature changes impacted the NV level (Fig. 5b) but in a weaker way than the MFI level (Fig. 5a). Moreover, the NV level was mostly affected at the beginning of the cell culture change (after 48 h of incubation or after replacing the cells for 48 h in a new environment), while it tended to go back to its initial value after 120 h in the same culture conditions. This observation could reflect the response time of each cell to environmental changes (Additional file 2).

We therefore concluded that temperature variations might well be responsible for the coordinated long-term variations that we previously observed (Fig. 2a).

### Indirect influences of temperature

We next ruled out the possibility that the observed variation in MFI was due to indirect effects of temperature.

First, we verified that temperature had no impact on the MFI of fixed cells (not shown) demonstrating that the observed effect was not due to a higher brilliance of an identical number of proteins.

We then checked whether or not the temperature could induce changes in either cell size or cells number. Cells were grown for 5 days at different temperatures and analyzed. Regarding the cell division rate, we did not notice any significant effect of the temperature on its value, as assessed by blue trypan counting (Additional file 3: Figure SD3B). The cell volume (as assessed by flow cytometry, see “Methods”, Additional file 3: Figure SD3A) was affected by the temperature: we noted an increase by 20 % of the cell size after 5 days at 35 °C and a decrease by 15 % at 39 °C.

One reason to check the effect of the temperature on the cell size and the cell growth rate was to be sure that the higher mCherry content measured at low temperature was not due to a reduction of the division rate and thus to an accumulation of the proteins in the cell. In such a case, we would notice a diminution of the growth rate and an increase of the cell size at 35 °C and the inverse would be true at 39 °C. Here, we do not notice any effect of temperature on the growth rate, which allows us rejecting this hypothesis. However, we do notice a size variation that is positively correlated with the overall in gene expression level. These two phenomena may therefore be part of the cellular response to the variation of temperature (see “Discussion” below).

Thus, the observed MFI variations are neither the consequence of a modification of the cell cycle, nor of intrinsic protein brilliance. It thus became reasonable to examine whether MFI variations may result from an effect of temperature on gene expression dynamics. We therefore examined how temperature changes could influence the fit of a two-state model of gene expression, which we previously demonstrated to account for fluorescent reporter gene expression distributions [9].

### Transcription burst sizes are systematically affected by temperature

**Fig. 6:**
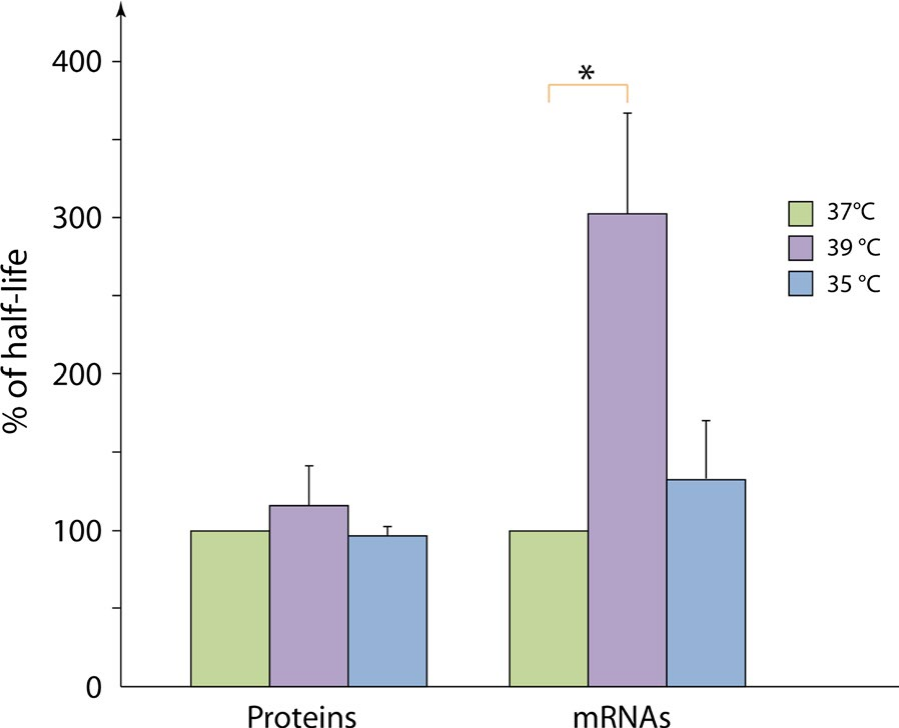
Impact of the temperature on the proteins and mRNA half-life. Cells were incubated during 3 days at 35, 37 and 39 °C and the mRNA and proteins half-life were determined as described in “Methods”. Shown is the ratio of the half-life at 35 or 39 °C divided by the half-life at 37 °C. N = 4, *error bars* represent SD, Wilcoxon test: *p value <0.05

The two-state model describes the protein production process as a three-step process: transcription (that depends on the gene on–off cycling), mRNA dynamics (production vs. degradation) and protein dynamics (production vs. degradation). It thus incorporates six parameters, three of which cannot be distinguished in the conditions considered here and are thus treated as an aggregated parameter (see “Methods”), resulting in a 4 parameters model. Among these parameters, two can be measured experimentally: the protein and mRNA half-life (respectively $1/\tilde{\rho }$ and $1/\tilde{\gamma }$). We therefore determined whether temperature variation was susceptible to influence their value. We observed that temperature had no influence on protein stability (Fig. 6). mRNA stability was differently affected: a significant threefold increase was detected at 39 °C, with no significant variation observed at 35 °C. Interestingly, this is exactly the opposite direction to what was expected from the observed lower expression at 39 °C. Other parameters of the expression process must therefore counterbalance the increase of RNA stability.

The two remaining parameters ($b$ and $f$) can be inferred from the experimental distributions of protein numbers. We previously showed [9] that in the considered system, these distributions derive from a protein production characterized by short and infrequent transcriptional events known as bursts. In such conditions the number of proteins produced during each burst ($b$) affects the horizontal scale of the distributions, while its shape is primarily affected by the bursts frequency ($f$, see Fig. 1). A suitable mathematical treatment (see “Methods”) allows to fit the experimental curves at the different temperatures, and to infer the underlying parameters.

As can be observed on the example shown on Fig. 1a, the scale of the curve is significantly modified (horizontal dilatation), while the shape remains comparable. In other words, each transcription burst produces fewer proteins at higher temperatures, in line with the reduction observed in the mean value of the protein distributions.

### The burst size depends on the insertion site, but its temperature dependence is universal

On Fig. 1b, we show the evolution of burst frequency and bursts size for different clones at different temperatures. The error bars reflect the heterogeneity among the sub-populations (a, b, c) of the same clone. We observed that the inferred burst size is reproducible for a given clone (main plot). On the opposite, the burst frequency differs between the clones and between temperatures, but also often between the sub-populations (large bars in the inset), maybe indicative of different modes of burst triggering depending on the conditions. A general tendency for the frequency to increase with temperature is observed (Spearman correlation 0.5, p value 0.003), but this result depends essentially on one experimental condition (1F4 at 39°). In two of the three clones (C5 and C11), the increase is not statistically significant (Spearman p values 0.60 and 0.11, vs. 0.006 for 1F4). Note that the frequency is also more difficult to fit, because the computation depends mostly on small values of intensity, which are mostly affected by background noise. Since we aim at identifying the generic, clone-independent effects, we did not further investigate the effect of temperature on burst frequency. In contrast, the burst size is significantly more variable among the clones than among the sub-population and it consistently decreases with temperature in all investigated clones (Spearman p-values ~2 × 10^−5^ for all clones). More precisely, we find that even though the burst size differs between the clones (different vertical values on the plot), the different lines are approximately parallel, indicating identical temperature dependence. In analogy to classical theories of reaction kinetics, this common slope has a physico-chemical signification, as the effective activation energy of the underlying Arrhenius law (see “Methods”). Here, the notion of “effective activation energy” refers to the fact that, strictly speaking, the activation energy is defined only for an elementary reaction process, and must therefore be taken with caution speaking of the molecular events associated to gene expression, which likely involve a complex network of individual step reactions [25, 26]. The measured slope corresponds to a value $E_{A} = - 60 \pm 3\;{\text{kcal}}/{\text{mol}}$ = $- 101 \pm 5k_{B} T$. Here the negative sign indicates that, in contrast to elementary processes which always get faster with $T$ (positive $E_{A}$), the dominant kinetic step here acts against protein production (see “Methods”). The common temperature dependence obtained for the three clones may thus reflect an identical chemical network for protein production in all cases, while the difference among the distributions suggest that the chromatin state at the different loci modulates how often and with which intensity the reporter gene is actively transcribed. In the next section, we discuss the possible mechanistic interpretations of these observations in more details.

## Discussion

In order to characterize the dynamics by which population heterogeneity in gene expression levels arises, we first isolated sub-fractions of cells from a clonal population and demonstrated the ergodic behavior of the system, with a very fast dynamics of less than 3 days for the largest fraction of cells. Numerous previous studies have reported the time dependence of the relaxation to the original distribution to be a matter of days (from 3 to 11 days, depending upon the cellular system involved; [15–19]). In our system, only the upper bound of the relaxation time could be determined, due to the very fast time scale involved. This confirms that in our cells the mechanisms generating short time heterogeneity operate at a rapid timescale (hours to days).

Moreover, in our experiment, even when the population was randomly separated in three sub-populations, these fractions kept similar MFI values at all timepoints along the experiment, indicating the importance of the genomic integration point in constraining the mean expression value.

We nevertheless observed that this mean expression value was highly variable along time for a given clone. Unexpectedly, those variations were correlated among all of our clones. This suggested the existence of an external factor acting on all our clones simultaneously. We showed that variations in CO_2_ concentration did not significantly modify the dynamic of the gene expression. In sharp contrast, an increase in temperature induced a large decrease in the MFI, and a decrease in temperature induced a strong increase in the MFI. Interestingly, the effect induced by the temperature seems to be reversible, as MFI tend to go back to their original values after replacing the cells at initial temperature. Such a reversibility of temperature effects was very similar to the effects we observed in a previous study when treating our cells with chromatin modifying drugs [2].

The observed variation in MFI is not due to a reduction in the growth rate of the cells. However, we could notice an increase of the cell size when the temperature is lower. We can propose that this phenomenon is a consequence of the higher protein production rate which would permit the cell to accommodate the accumulation of a higher protein amount.

These experiments were performed on 6C2 cells, a chicken erythroblast cell line transformed by the avian erythroblastosis virus [20]. Since the chicken body temperature is 41 °C, one can rule out that our cells were experiencing a heat shock at any temperature tested. For similar studies to be conducted in mammalian cells, it is therefore probable that the temperature range should be adjusted, in order not to bypass the standard temperature of 37 °C.

The culture temperature therefore seems to be an important factor to take into account when analyzing gene expression. One should also emphasize that the NV was only poorly affected, which is in line with our molecular explanation (see below).

We showed that temperature affects the number of proteins produced during each transcription burst, and we quantified this dependence for cells incubated for 120 h (Fig. 1). Unsurprisingly, we found that cells incubated only 48 h exhibited a smaller temperature dependence (~25 % weaker slope), and the observed effect was even smaller for cells that were first cultured for 120 h, then set back to 37 °C for 48 h (~70 % weaker slope). These observations are related to the lifetime of the fluorescent protein employed (around 47 h): in these samples, a significant fraction of the proteins were in fact produced at a temperature of 37 °C, which results in diluting the effect under investigation. In the 120 h samples, although a minor remaining proportion of proteins was produced before the temperature change, the quantified temperature dependence reflects much more faithfully the underlying molecular processes.

We investigated what could be the molecular basis for such a temperature-induced effect. We first ruled out a possible influence of temperature on the stability of the reporter mRNAs or proteins. Indeed temperature had either no effect or an inverse effect on their stability. An increase of the mRNA stability while a diminution in proteins is observed could reflect an impact of the temperature on the protein translation rate. However, the literature seems quite unconvincing as depending on the study or possibly on the organism, the temperature does not display the same impact on protein translation rate [27, 28].

We therefore tried to determine whether temperature could alter the dynamics of gene expression, by fitting our data to a two-state model. We established that the burst frequency could be increased by temperature, but this effect was observed in only one of the three clones tested, and thus depends on the transgene insertion point. Since the effect of the temperature on the mean gene expression was observed within all three clones tested, the modification in burst frequency is likely not to be the dominant underlying mechanism. In contrast, the burst size inferred from the data, i.e. the number of proteins produced during each burst, was found to decrease with temperature independently of the transgene insertion point, in line with the reduction observed in the mean value of the protein distribution. A symmetrical situation was observed for lower temperatures. These results therefore suggest that, at least for the investigated promoter, temperature modifies the gene expression by modulating the mean burst size.

One may then wonder what molecular processes are driving this modulation. As previously mentioned, the burst process likely involves a complex combination of elementary processes [25], which could a priori all be influenced by temperature, either directly or indirectly. Some of these processes might be specific to the gene/locus considered: for instance, any event or compound modifying epigenetic marks such as histone acetylation or DNA methylation, or nucleosome positioning, will affect chromatin condensation and subsequently also the gene expression dynamics. But temperature might also be involved in a more global control of gene expression, involving e.g., the metabolic pathways, which would then affect all genes. Based on our data, can we infer how these different layers of regulation are affected by temperature variations ?

In the framework of the two-state model, the burst frequency is given by the chromatin opening rate, i.e. a parameter that depends on the insertion point of the transgene [9]. Interestingly, previous works on plants subjected to large temperature fluctuations (10 °C) [29] have highlighted a complex control of gene expression by temperature involving a regulation of the chromatin state. The clone-specific effect of temperature on burst frequency inferred from our data might reflect a comparable mode of control in our mammalian system, which would influence the duration between two bursting events. If this control exists, its efficiency seems extremely dependent on the genomic location.

The mean burst size, on the other hand, depends on several reaction rates (see equation in “Methods”): it increases with the mRNA and protein production rates, and decreases with the RNA degradation rate and the chromatin closing rate (1/burst duration). Since the RNA degradation rate was shown to decrease with temperature (at 39 °C), it cannot account for the observed decrease in burst size. Rather, this feature could result from a decrease in either burst intensity (RNA/protein production rate) or in burst duration. Our data lacks the required time resolution to discriminate between these alternate explanations; this may be addressed in the future by single-cell time-lapse microscopy using short-lived proteins [30]. However, the observation of a common temperature dependence of the mean burst size in all our clones (even though the value of the burst size is different), tends to suggest an effect independent of the insertion point. This seems to favor the hypothesis of an effect of temperature on burst intensity, driven by a reduction in RNA or protein production rate, rather than a burst duration (related to local chromatin dynamics). With our study involving only one reporter gene, we cannot distinguish whether this effect is gene-specific or reflects a more global response to temperature variations. In the latter case, a plausible attractive explanation is that the cell adjusts its metabolism to temperature variations, which would in turn modify the efficiency of the transcription/translation machinery in a gene-nonspecific manner. This suggestion is supported by the positive correlation between temperature variations and metabolic activity observed in a wide range of organisms [31], and secondly by the role of metabolic fluxes in coupling metabolic control and gene expression [32–34], which may be involved in cell-decision making processes involved in cell differentiation [35]. Maybe surprisingly, in our case, the increased metabolic activity driven by higher temperature would then result in a reduction in gene expression.

In this work, we have underlined that small variation in cell culture temperature significantly alters the transcriptional process of the CMV promoter. Since CMV is one of the strongest promoters and, as a consequence, is often used in studies requiring transient or stable transgene expression, our study emphasizes that the cell culture temperature should then be tightly controlled to avoid misinterpretation of the data. In our case, using such an exogenous system allowed us to directly relate the protein distributions observed to the transcriptional process.

The next step should now be dedicated to the impact of temperature variations in the expression of endogenous genes, in order to assess the generality of our observations and to analyze the underlying molecular mechanisms mentioned above. Regarding this question, the reader should note that a putative global increase or decrease of expression related to temperature variations cannot be assessed by usual transcriptomics techniques (e.g. microarray or RNA sequencing), which only provide expression levels normalized within an experimental condition (see e.g. [36]). A new study will thus require specific experimental setups and a dedicated analysis methodology, so as to provide absolute levels of transcripts per cell. This remark could be an explanation why an effect as general as the one that we observe, and which might have drastic consequences in a wide range of experiments, has to our knowledge never even addressed in the literature, even though many studies were dedicated to the relative expression changes induced by temperature variations (see e.g. [37] or [38]). Even though our results are in principle limited to a single exogenous promoter, they suggest that similar effects might be present in a wide range of experiments, but remain undetected in absence of a dedicated methodology. Conversely, the latter will allow exploring the physiological context where temperature could be involved in gene expression modifications in particular in the circadian rhythm. It has been shown that peripheral clocks are entrained by temperature variations of small amplitude (2.5 °C) in homeothermic vertebrates [39]. It is not presently known if this involves transcriptional regulation or not. The most obvious circumstance in metazoans were such a temperature-dependent process might be relevant is of course episodes of fever; here, we note that the promoter used in our study comes from a virus which may benefit from an adjustment to fever. Further work would particularly gain from newly developed single-cell techniques (see e.g. [40]) that may give access to the mean and NV of endogenous genes expressed in cells of the immune system when confronted to a sudden elevation of temperature.

## Conclusion

We investigated the sources of gene expression stochasticity by using a cell line expressing a fluorescent reporter gene under the control of a CMV promoter. We observed that the mean expression value was highly variable along time for a given clone, but that those variations were strongly correlated among all of our clones. We found that small temperature differences could account for such an effect since 2 °C variations were shown to significantly affects the mean expression of our reporter gene. We further demonstrated that temperature acts by modifying the size of transcription bursts, while the burst frequency of the investigated promoter is less systematically affected.

We therefore report, for the first time, that transcription burst size is a key parameter for gene expression that metazoan cells from homeotherm animals can modify in response to an external thermal stimulus.

This is an intriguing observation that raises the question as to whether it is specific to the system used (CMV promoter in chicken cells) or whether this is a more generic phenomenon.

## Additional files

10.1186/s12867-015-0048-2 Mean of Fluorescence Intensity (MFI) variability between the 3 sub-populations of each clone during time. The MFI of 3 clones, C5 (A), C11 (B) and 1F4 (C), randomly separated in 3 sub-populations in day 0, had been regularly measured by flow cytometry on 50,000 gated lived cells during 230 days.
10.1186/s12867-015-0048-2 Normalized Variance (NV) variability between the 3 sub-populations of each clone during time. At day 0, each clone C5 (A), C11 (B) and 1F4 (C) had been randomly separated in 3 sub-populations and then, the NV were regularly assessed by flow cytometry on 50,000 gated lived cells during 230 days.
10.1186/s12867-015-0048-2 Impact of the temperature on the cell size (A) and the cell division rate (B). The cells were incubated at 3 different temperatures, 35°C, 37 °C (standard temperature) and 39°C during 5 days. Then, the cell size was determined by flow cytometry (A) and the cell division rate was determined by a blue trypan counting (B).
