# Erratum to: Temperature-induced variation in gene expression burst size in metazoan cells

**DOI:** 10.1186/s12867-015-0054-4

**Published:** 2016-02-03

**Authors:** Ophélie Arnaud, Sam Meyer, Elodie Vallin, Guillaume Beslon, Olivier Gandrillon

**Affiliations:** Centre de Génétique et de Physiologie Moléculaire et Cellulaire (CGPhiMC), CNRS UMR5534, Université de Lyon, Université Lyon 1, 69622 Lyon, France; Laboratoire d’InfoRmatique en Image et Systèmes d’information (LIRIS), CNRS UMR5205, INSA-Lyon, INRIA, Université de Lyon, 69621 Lyon, France; Inria Team Dracula, Inria Center Grenoble Rhône-Alpes, Montbonnot-Saint-Martin, France; Inria Team Beagle, Inria Center Grenoble Rhône-Alpes, Montbonnot-Saint-Martin, France; Division of Genomic Technologies, RIKEN Center for Life Science Technologies, Yokohama, Japan; INSA-Lyon, CNRS UMR5240 Microbiologie, Adaptation et Pathogénie, Université de Lyon, 69622 Lyon, France; Laboratoire de Biologie Moléculaire de la Cellule, Ecole Normale Supérieure de Lyon, CNRS, Université de Lyon, 46 Allée d’Italie, 69007 Lyon, France

## Erratum to: BMC Molecular Biol (2015) 16:20 DOI 10.1186/s12867-015-0048-2

Unfortunately, the original version of this article [1] contained two mistakes. The figure order was incorrectly captured during the production process with Figure 1 intended as Figure 6. All other figures are renumbered to accommodate this. The reference to the incorrect Figure 1 in the text should also have read, “(2) the RNA half-life, and it is further validated by the good agreement of the model with experimental curves (see below).”

The original version of the article has been updated to rectify these errors.
